# Flow‐controlled expiration improves gas exchange in anaesthetised horses undergoing orthopaedic surgery

**DOI:** 10.1111/evj.70079

**Published:** 2025-08-21

**Authors:** Klaus Hopster, Joao Henrique Neves Soares, David Levine, Kyla Ortved, Bernd Driessen, Joaquin Araos

**Affiliations:** ^1^ Department of Clinical Studies—New Bolton Center School of Veterinary Medicine, University of Pennsylvania Philadelphia Pennsylvania USA; ^2^ Department of Radiological and Surgical Sciences School of Veterinary Medicine at University of California, Davis Davis California USA; ^3^ Department of Clinical Sciences College of Veterinary Medicine, Cornell University Ithaca New York USA

**Keywords:** compliance, gas exchange, horse, oxygenation, V/Q index

## Abstract

**Background:**

Flow‐controlled expiration (FLEX) has been shown to significantly enhance oxygenation in horses under laboratory conditions.

**Objective:**

This study aims to corroborate these findings by evaluating the effects of FLEX on gas exchange in a randomised clinical trial involving a large population of clinical horses undergoing orthopaedic surgery.

**Study Design:**

Prospective randomised clinical trial.

**Methods:**

A total of 406 healthy adult horses scheduled for elective orthopaedic procedures were recruited for this prospective clinical trial. Horses were randomly assigned to FLEX or VCV (volume‐controlled ventilation) groups in dorsal (VCV‐D and FLEX‐D) or lateral recumbency (VCV‐L and FLEX‐L). Arterial blood gases were measured at 30, 75, and 120 min post‐induction to assess arterial oxygenation (arterial partial pressure of oxygen to inspired fraction of oxygen ratio, PaO_2_/FiO_2_). A global index of ventilation/perfusion matching ([PaCO_2_ − ETCO_2_]/PaCO_2_) was also calculated. Peak airway pressure (P_peak_) and tidal volume were measured to calculate dynamic respiratory system compliance (Cdyn). Data were compared with repeated‐measures ANOVA.

**Results:**

Horses ventilated with FLEX showed significantly higher PaO_2_/FiO_2_ (FLEX‐D vs. VCV‐D, 369 ± 42 vs. 198 ± 112 mmHg, *p* < 0.001; FLEX‐L vs. VCV‐L, 436 ± 38 vs. 249 ± 88 mmHg, *p* < 0.001). FLEX also improved Cdyn (FLEX‐D vs. VCV‐D, 0.81 ± 0.1 vs. 0.64 ± 0.12, *p* = 0.01) and the global V̇/Q̇ index ([PaCO_2_ − ETCO_2_]/PaCO_2_) (FLEX‐D vs. VCV‐D, 0.11 ± 0.03 vs. 0.18 ± 0.03, *p* = 0.03) in dorsal‐positioned but not lateral‐positioned horses.

**Main Limitations:**

Anaesthesia protocols were not standardised; anaesthetists were not masked to the intervention of interest, and findings may not be generalisable to other patient populations.

**Conclusions:**

These results confirm previous laboratory findings, demonstrating that FLEX improves oxygenation, ventilation–perfusion matching, and respiratory mechanics compared to VCV in a large clinical population of anaesthetised horses.

## 1 INTRODUCTION

Equine general anaesthesia remains associated with a higher mortality rate than that observed in humans and most other species.^1^, ^2^ This may be partly attributable to cardiopulmonary pathophysiological changes, such as hypoxaemia and hypotension, which are especially common during general anaesthesia in dorsal recumbency.^3^ The substantial weight of the horse's abdominal viscera on the diaphragm increases the risk of hypoxaemia, primarily by causing compression atelectasis in non‐aerated lung regions.^4^, ^5^ In dorsal recumbency, the dependent (dorsal) lung regions are preferentially perfused but prone to collapse due to abdominal pressure, leading to marked ventilation–perfusion (V̇/Q̇) mismatch.^6^, ^7^ Intraoperative hypoxaemia and lung collapse are associated with prolonged intensive care and hospital stays, increased respiratory failure, and pneumonia rates in humans.^8^, ^9^ Similarly, in horses, these conditions have been linked to postoperative complications, including poor anaesthetic recovery quality.^10^ Therefore, optimising ventilation strategies during equine general anaesthesia is critical to minimise intra‐ and postoperative complications.

Various ventilation strategies, including alveolar recruitment manoeuvres followed by sustained positive end‐expiratory pressure (PEEP), have demonstrated improvements in gas exchange in anaesthetised horses.^11^, ^12^, ^13^, ^14^, ^15^ However, the high peak airway pressures needed to maintain lung recruitment may contribute to ventilator‐associated lung injury, even in healthy subjects, by exacerbating regional stress and strain.^13^, ^16^ Excessive PEEP can also compromise cardiovascular function by reducing venous return to the heart, resulting in lower stroke volume and cardiac output.^14^, ^17^

Recent attention has turned towards optimising the expiratory phase of ventilation to enhance lung function.^18^, ^19^, ^20^, ^21^, ^22^ Flow‐controlled expiration (FLEX), a novel ventilation method to provide volume‐controlled ventilation (VCV), modifies the otherwise passive expiratory phase by limiting initial high expiratory flow and maintaining gas flow throughout expiration. In porcine models of acute respiratory distress syndrome, FLEX reduced ventilation‐induced lung injury, decreased pulmonary oedema, enhanced dynamic compliance, and improved gas exchange.^18^ Furthermore, FLEX redistributed ventilation towards dorsal lung regions in both healthy and injured lungs.^19^ It has been recently shown that in research horses, FLEX improves regional matching of ventilation and perfusion by promoting a more uniform and sustained lung emptying during expiration. This resulted in significant increases in oxygenation, gas exchange and respiratory system mechanics when compared with conventional VCV.^20^, ^21^, ^22^

This study aims to validate findings from highly controlled experiments in research horses in a larger clinical population. We hypothesised that horses ventilated with FLEX would exhibit improved gas exchange, indicated by enhanced indices of pulmonary gas exchange, regardless of recumbency.

## 2 MATERIALS AND METHODS

**FIGURE 1:**
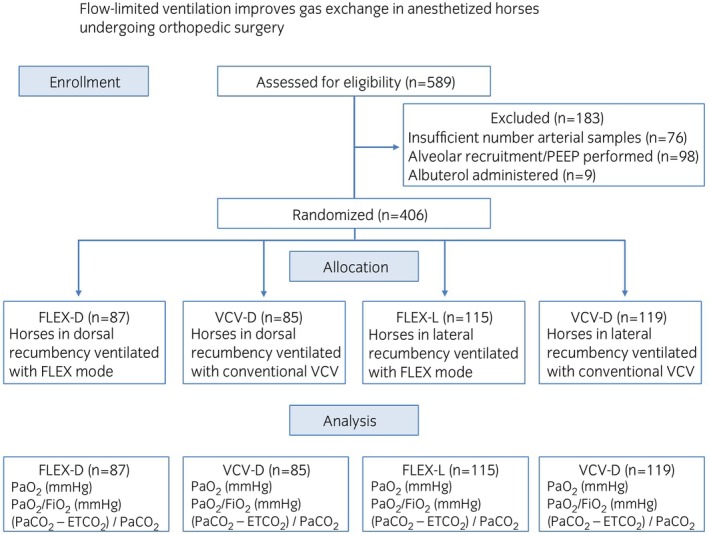
CONSORT flowchart. This flowchart illustrates the progress of participants through each stage of the study evaluating the effect of FLEX ventilation on oxygenation in horses during elective surgery. It outlines the recruitment, allocation, and analysis of the study population, in accordance with the CONSORT guidelines.

This study was designed as a prospective, non‐blinded, single‐centre clinical trial, and the study adhered to the ARRIVE guidelines.^23^ Data were collected from the anaesthesia records of healthy adult horses (ASA status I and II) undergoing general anaesthesia for elective orthopaedic surgical procedures in both dorsal and lateral recumbency between February 2022 and February 2024 (Figure 1, Table S1); they were anaesthetised by three board‐certified veterinary anaesthesiologists and two anaesthesia residents under their direct supervision. Recumbency was determined based on surgical requirements and was not randomised. All enrolled horses were determined to be systemically healthy based on physical examination and pre‐anaesthetic bloodwork, including determination of packed cell volume, total solids, fibrinogen, and a complete blood count. Further inclusion criteria: Horses between 3 and 19 years old with a bodyweight of at least 380 kg; horses had to be anaesthetised for at least 120 min without receiving alveolar recruitment or PEEP ventilation during the study period. Further, at least three arterial blood samples had to be taken at timepoints 25–35 min, 65–75 min, and 110–120 min after initiation of mechanical ventilation.

Patients were fasted for at least 6 h before general anaesthesia but had unrestricted access to water. On the day of surgery, a 14‐gauge catheter was placed into the left or right jugular vein. Animals received either flunixin meglumine or phenylbutazone and antimicrobials if the procedure required it. They were pre‐medicated with intravenous (IV) acepromazine (0.03 mg/kg, Covetrus North America; Acepromazine Maleate® 10 mg/mL) and xylazine (to effect, 0.6–0.9 mg/kg; Bayer Healthcare, LLC; Rompun® 100 mg/mL), and general anaesthesia was induced with IV ketamine (2.2 mg/kg; MWI/VetOne; Zetamine™ Injection 100 mg/mL) and midazolam (0.05 mg/kg; West‐Ward, Inc.; Midazolam HCl 50 mg/mL). The horses' tracheas were orotracheally intubated with an appropriately sized endotracheal tube and subsequently hoisted and positioned on the surgical table in either lateral or dorsal recumbency, depending on the recumbency required for the surgical procedure. The endotracheal tube was connected to a large animal anaesthetic circle system and the horses were fitted with standard anaesthesia monitoring equipment (Tafonius Large Animal Anesthesia Workstation, Hallowell EMC). Standard of care monitoring was performed including electrocardiogram via base‐apex lead and pulse oximetry (SpO_2_) using a probe positioned on the tongue of the horses. A side‐stream gas analyser was used to measure the end‐tidal partial pressure of CO_2_ (ETCO_2_) and the end‐tidal concentration of inhalant anaesthetics by the infrared technique and the inspired concentration of oxygen (FiO_2_) was measured by a paramagnetic sensor. The gas analyser was calibrated daily using room air and monthly using standard calibration gas mixtures according to the manufacturer's instructions. The sampling port of the gas analyser was positioned at the wye piece of the breathing system. A 20‐gauge catheter was placed into the facial artery and connected to a heparinised saline system with a transducer for the measurements of systolic (SAP), mean (MAP) and diastolic (DAP) arterial pressures and for the collection of blood samples for blood gas analysis. The blood pressure transducer was calibrated and zeroed at atmospheric pressure. It was positioned at the level of the right atrium, estimated by a horizontal line drawn from the point of the shoulder (greater tubercle of the humerus) perpendicular to the floor. For horses positioned in lateral recumbency, the transducer was placed at the level of the withers.

General anaesthesia was maintained with isoflurane (Henry Schein, Inc.; Isoflurane USP) or desflurane (Baxter International, Inc.; Suprane USP) in oxygen; dobutamine (Hospira, Inc.; DOBUTamine HCl) constant rate infusion was administered as necessary to maintain MAP >70 mmHg. Intravenous crystalloid solutions (Baxter International, Inc.; Plasmalyte A) were infused at a rate of 3–4 mL/kg/h.

Prior to anaesthesia, horses were randomly assigned to either conventional VCV or FLEX using computer‐generated random numbers. The lungs were mechanically ventilated immediately after positioning on the surgery table. Specifically, for horses assigned to FLEX, the V_T_ release was set at 98%, meaning that the linearised exhalation of V_T_ occurred over 98% of the expiratory time, with expiratory flow reaching zero only in the final 2% of expiration (Figure S1).

For all horses, ventilation was adjusted to deliver a V_T_ of 14 mL/kg at an inspiratory to expiratory time (I:E) ratio of 1:2 and an initial rate of 6 breaths per minute; this was adjusted if needed to ensure ETCO_2_ in the range of 35–55 mmHg.^20^, ^22^ To isolate the effect of FLEX on the proposed outcomes, PEEP was set at 0 cmH_2_O for all horses.

Arterial blood samples were obtained in pre‐heparinised syringes at 25–35 (timepoint T1), 65–75 (T2) and 110–120 (T3) min after initiation of mechanical ventilation and analysed immediately using a blood gas analyser (Opti Medical Systems; Opti CCA‐TS2 Blood Gas and Electrolyte Analyser) to measure the arterial partial pressure of oxygen (PaO_2_) and the arterial partial pressure of carbon dioxide (PaCO_2_). The PaO_2_/FiO_2_ ratio was calculated for each timepoint of data collection as an index of oxygenation. A global V̇/Q̇ index was also calculated for each timepoint as (PaCO_2_ − ETCO_2_)/PaCO_2_. The dynamic compliance (Cdyn) was calculated as a mean value over 5 breaths using the following equation:

$$\text{Cdyn}=\left(P_{\text{peak}}–\text{PEEP}\right)/\mathrm{TV}$$

At the end of surgery, horses were disconnected from the anaesthetic circuit and moved to a recovery stall. During the recovery period, the endotracheal tube was replaced with a nasotracheal tube used to insufflate oxygen at a rate of 15 L/min, and all horses received intravenous xylazine (0.1–0.25 mg/kg IV) or dexmedetomidine (1–2 μg/kg IV) for sedation. Recovery was assisted using head and tail ropes following institutional protocol. All horses recovered well and showed no general anaesthesia‐related complications within the first 24 h after surgery.

## 3 DATA ANALYSIS

Data were analysed using the statistical software SAS 9.3 and GraphPad Prism Version 7. Visual assessment of QQ‐plots and the Shapiro–Wilk test was used to confirm the normal distribution of model residuals of dependent variables and demographic data such as age and bodyweight.

No formal sample size calculation was conducted due to the exploratory and opportunistic nature of the study.

For analysis, horses were assigned to four groups: animals positioned in dorsal recumbency and ventilated using FLEX (FLEX‐D), animals positioned in dorsal recumbency and ventilated using conventional VCV (VCV‐D), animals positioned in lateral recumbency and ventilated using FLEX (FLEX‐L), and animals positioned in lateral recumbency and ventilated using conventional VCV (VCV‐L). Primary outcomes included the PaO_2_/FiO_2_ and the (PaCO_2_ − E_T_CO_2_)/PaCO_2_. Secondary outcomes included Cdyn, as well as cardiovascular variables such as MAP and heart rate, as well as mean dobutamine and volatile anaesthetic requirements. Outcomes were analysed and compared as total mean value over the first 120 min of general anaesthesia.

Variables collected at the time points T1, T2, and T3 were compared between ventilation modes (i.e., FLEX vs. VCV) in horses at the same recumbency (i.e., FLEX‐D vs. VCV‐D) using a two‐factorial variance analysis for repeated measurements and Bonferroni correction for multiple comparisons. Mauchly's test of sphericity was performed, and Greenhouse–Geisser corrections were applied when the assumption of sphericity was violated. No major violations or influential outliers were identified during residual diagnostics.

The level of significance was set to 5% (*p* < 0.05).

## 4 RESULTS

A total of 589 horses were assessed for eligibility. A total of 98 animals were excluded because PEEP was initiated and/or an alveolar recruitment was performed (89 animals ventilated with VCV and 9 animals ventilated with FLEX) and 9 horses were excluded because albuterol was administered (all VCV).

A total of 406 horses fit the inclusion criteria (Figure 1, Table 1) and were enrolled in this study. These animals were between 3 and 16 years of age with weights ranging from 364 to 610 kg.

**TABLE 1:** Number of animals positioned in dorsal (D) or lateral (L) recumbency and ventilated using either conventional VCV (VCV) or FLEX ventilation (FLEX) and their mean bodyweight (kg) and mean age (years).

	FLEX—D	VCV—D	FLEX—L	VCV—L
Number of horses	87	85	115	119
Weight (±SD) (kg)	477 (±26)	452 (±37)	485 (±31)	509 (±24)
Age (±SD) (years)	5 (±4)	6 (±5)	5 (±4)	7 (±4)

**TABLE 2:** Mean (±SD) arterial blood pressure (MAP), heart rate (HR), dobutamine requirements (DOB) and median (range) volatile anaesthetic exposure in MAC hours (MAC‐h) as well as isoflurane versus desflurane anaesthetised patient numbers over the first 120 min of general anaesthesia in horses positioned in dorsal (D) or lateral (L) recumbency and ventilated using either conventional VCV (VCV) or FLEX ventilation (FLEX) modes.

	FLEX—D	VCV—D	FLEX—L	VCV—L
MAP (mmHg)	75 ± 11	78 ± 9	77 ± 13	76 ± 8
HR (bpm)	38 ± 5	36 ± 7	31 ± 4	32 ± 8
DOB (μg/kg/min)	0.75 ± 0.12	0.70 ± 0.09	0.55 ± 0.15	0.65 ± 0.08
Volatile anaesthetic exposure (MAC‐h)	0.9 (0.7–1.1)	0.9 (0.7–1.2)	0.9 (0.8–1.1)	1.0 (0.8–1.2)
Isoflurane versus desflurane used	55 versus 32	57 versus 28	78 versus 37	81 versus 38

There were no differences in age (*p* > 0.4) or bodyweight (*p* > 0.6) (Table 1) or in MAP (*p* > 0.8), heart rate (*p* > 0.6), and dobutamine dose administered (*p* > 0.3) or volatile anaesthetic dose requirements (*p* > 0.6) (analysed as total mean over the first 120 min of general anaesthesia) between groups (Table 2).

The PaO_2_ and the PaO_2_/FiO_2_ ratio were significantly higher in horses assigned to group FLEX‐D vs. VCV‐D and also significantly higher in horses assigned to group FLEX‐L vs. VCV‐L at all three time points (*p* < 0.001) (Table 3). Horses of group FLEX‐D had significantly better Cdyn at T1 (*p* = 0.002), T2 (*p* = 0.001), and T3 (*p* = 0.001) compared to animals ventilated with VCV‐D (Table 3). Further, these horses had significantly lower PaCO_2_ − E_T_CO_2_/PaCO_2_ ratios at T1 (*p* = 0.027), T2 (*p* = 0.035), and T3 (*p* < 0.001) compared to horses in group VCV‐D (Table 3). Animals positioned in lateral recumbency showed no difference in the (PaCO_2_ − E_T_CO_2_)/PaCO_2_ ratio at any time point between ventilation modes. There was no difference in oxygenation and ventilation variables between horses in dorsal and lateral recumbency when ventilated using the same ventilation mode. All horses survived to discharge, and no respiratory complications were reported during the 24‐h postoperative monitoring period.

**TABLE 3:** Mean and standard deviation of PaO_2_ (mmHg), PaO_2_/FiO_2_ ratio (mmHg), PaCO_2_ (mmHg), E_T_CO_2_/PaCO_2_ ratio and peak airway pressure (PIP, cmH_2_O) in horses positioned in dorsal (D) or lateral (L) recumbency and ventilated using either conventional VCV (VCV) or FLEX ventilation (FLEX).

	FLEX—D			VCV—D		
	T1	T2	T3	T1	T2	T3
PaO_2_ (mmHg)	369 ± 42^**c**^	395 ± 58^**c**^	421 ± 55^**c**^	198 ± 112^**c**^	171 ± 73^**c**^	164 ± 68^**c**^
PaO_2_/FiO_2_ (mmHg)*	402 ± 45^**c**^	420 ± 61^**c**^	443 ± 85^**c**^	212 ± 123^**c**^	181 ± 78^**c**^	174 ± 71^**c**^
PaCO_2_ (mmHg)	46 ± 3	45 ± 4	45 ± 4	44 ± 5	43 ± 6	45 ± 4
(PaCO_2_ − E_T_CO_2_)/PaCO_2_*	0.12 ± 0.02^**a**^	0.11 ± 0.03^**a**^	0.09 ± 0.02^**b**^	0.18 ± 0.03^**a**^	0.18 ± 0.03^**a**^	0.19 ± 0.03^**b**^
Cdyn (mL/kg/cmH_2_O)	0.79 ± 0.11^**b**^	0.82 ± 0.11^**b**^	0.83 ± 0.09^**b**^	0.67 ± 0.14^**b**^	0.64 ± 0.12^**b**^	0.68 ± 0.11 ^**b**^
PIP (cmH_2_O)	17 ± 2^a^	17 ± 2^b^	17 ± 3^b^	20 ± 2^a^	21 ± 3^b^	20 ± 3^b^

	FLEX—L			VCV—L		
	T1	T2	T3	T1	T2	T3
PaO_2_ (mmHg)	436 ± 38^**c**^	452 ± 61^**c**^	448 ± 49^**c**^	249 ± 88^**c**^	224 ± 91^**c**^	197 ± 76^**c**^
PaO_2_/FiO_2_ (mmHg)*	479 ± 49^**c**^	480 ± 64^**c**^	471 ± 89^**c**^	273 ± 96^**c**^	238 ± 97^**c**^	206 ± 80^**c**^
PaCO_2_ (mmHg)	42 ± 4	43 ± 4	43 ± 5	43 ± 5	46 ± 6	45 ± 5
(PaCO_2_ − E_T_CO_2_)/PaCO_2_*	0.10 ± 0.02	0.09 ± 0.02	0.09 ± 0.02	0.15 ± 0.03	0.15 ± 0.03	0.13 ± 0.03
Cdyn (mL/kg/cmH_2_O)	0.88 ± 0.07	0.89 ± 0.09	0.91 ± 0.08	0.87 ± 0.11	0.86 ± 0.13	0.86 ± 0.1
PIP (cmH_2_O)	16 ± 2	16 ± 2	15 ± 3	16 ± 3	17 ± 3	16 ± 3

*Note*: Arterial blood samples were obtained at 25–35 min (timepoint T1), 65–75 min (timepoint T2) and 110–120 min (timepoint T3) after initiation of mechanical ventilation. Primary statistical outcomes include PaO_2_/FiO_2_ ratio and the (PaCO_2_ − ETCO_2_)/PaCO_2_ ratio and are marked with an asterisk, secondary outcomes include Cdyn and PIP. Significant differences (*p* < 0.05) based on repeated‐measures ANOVA with Bonferroni correction for multiple comparisons. Between animals in similar recumbency when ventilated using FLEX or VCV at the same time point are marked as follows: a = *p* < 0.05–0.01, b = *p* < 0.01–0.001, c = *p* < 0.001.

## 5 DISCUSSION

Our study was designed to evaluate the effects of FLEX ventilation on indices of pulmonary gas exchange in horses within a large clinical setting. Horses ventilated using FLEX had a significantly higher PaO_2_ and PaO_2_/FiO_2_ ratio, better Cdyn and lower global V̇/Q̇ index when compared to horses ventilated using conventional VCV mode, indicating improved pulmonary function during FLEX ventilation. These findings demonstrated that FLEX ventilation supports pulmonary gas exchange and mechanics more effectively than conventional VCV in anaesthetised healthy horses undergoing orthopaedic surgery.

FLEX ventilation, as a novel mode of mechanical ventilation, improves the PaO_2_ and the PaO_2_/FiO_2_ ratio in healthy, anaesthetised horses by addressing critical aspects of equine pulmonary mechanics and gas exchange. Unlike traditional ventilation modes that rely on passive exhalation, FLEX introduces a controlled exhalatory phase, which regulates the flow rate during exhalation. This mechanism mitigates airway collapse and promotes uniform alveolar emptying, particularly in dependent lung regions, thereby enhancing alveolar ventilation.^19^, ^20^, ^24^ Horses under general anaesthesia are particularly prone to atelectasis formation and V̇/Q̇ mismatch due to their large body mass, the anatomic shape of the diaphragm, recumbency, and the effects of anaesthetic agents on diaphragmatic function and respiratory system compliance.^4^, ^6^, ^25^, ^26^ By reducing the likelihood of alveolar collapse, FLEX ventilation maintains better aeration of dependent lung zones, improving pulmonary gas exchange, as reported in the horses of this study anaesthetised in dorsal or lateral recumbency.

FLEX ventilation optimises the expiratory flow profile, thereby preventing premature airway closure and minimising dynamic airway compression during exhalation.^19^, ^22^, ^24^ Secondly, FLEX ventilation may increase functional residual capacity, a key determinant of gas exchange efficiency.^20^ By maintaining more open alveoli at the end of exhalation, FLEX reduces intrapulmonary shunting and facilitates better matching of ventilation and perfusion.^20^, ^22^

While no haemodynamic differences were observed in this study, FLEX has been shown to be cardiovascularly sparing; previous laboratory studies have demonstrated haemodynamic stability during FLEX ventilation. Therefore, by preserving haemodynamics while significantly improving arterial oxygenation, FLEX may enhance oxygen delivery to tissues compared to VCV.

Another contributing factor may be the reduction of hyperinflation or over‐distension in non‐dependent lung regions. Traditional ventilatory modes, particularly those with high tidal volumes or high end‐expiratory pressure ventilation, can lead to hyperinflation of non‐dependent alveoli, potentially inducing ventilator‐induced lung injury (VILI) or exacerbating V̇/Q̇ mismatch.^27^, ^28^ Controlled expiratory flow during FLEX ventilation may minimise these risks, distributing ventilation more evenly across the lung fields and reducing strain on the pulmonary parenchyma.^20^

The FLEX ventilated horses had not only higher PaO_2_/FiO_2_ ratios indicating improved V̇/Q̇ matching but also a lower global V̇/Q̇ index commonly referred to as alveolar dead space. This index is calculated using PaCO_2_ instead of mean PACO_2_ and therefore is also affected by any changes in venous admixture and right‐to‐left shunt.^29^ Consequently, the lower values of global V̇/Q̇ index observed during FLEX ventilation can suggest a decrease in alveolar dead space when compared to conventional VCV. However, the higher PaO_2_/FiO_2_ observed during FLEX ventilation can also be responsible for the lower global V̇/Q̇ index. The fact that FLEX improved the ventilation of dependent lung regions as well as the perfusion of the non‐dependent lung regions in horses supports the suggestion that this ventilation mode decreases alveolar dead space (high V̇/Q̇) in addition to minimising venous admixture/shunt (low or zero V̇/Q̇).^20^

These findings, while observed in healthy horses, are particularly promising for clinical applications in a wider population of anaesthetised large animals.

Similar improvements in gas exchange and respiratory mechanics with FLEX ventilation have been reported in swine models of acute lung injury and in human studies involving patients under general anaesthesia.^18^, ^19^, ^24^ In porcine studies, FLEX reduced VILI and pulmonary oedema, enhanced dynamic compliance, and improved dorsal lung aeration—mechanistic findings that align with our observations in horses.^18^, ^24^

Notably, while horses in dorsal recumbency benefitted markedly from FLEX in terms of improvement of a global index of ventilation/perfusion matching, the effect was not as prominent in horses placed in lateral recumbency. This may be due to anatomical or gravitational differences in perfusion distribution. Alternatively, this could represent a type II error (β‐error), as the effect size in this subgroup may be smaller than in dorsal recumbency. Further studies are warranted to confirm this finding. In contrast, swine and human studies often report more uniform improvements in V̇/Q̇ matching across positions.^28^ This discrepancy may reflect species‐specific differences in lung anatomy and perfusion gradients. For example, equine lungs are particularly susceptible to dorsal atelectasis formation due to their large mass, the anatomic shape of their diaphragm, and gravitational compression when recumbent.^6^, ^25^ In contrast, lateral positioning in horses redistributes perfusion more heterogeneously,^6^ potentially limiting the consistent benefit of FLEX in this posture.

Our findings also complement those of Douglas et al.,^30^ who evaluated FLEX using a piston ventilator in healthy horses and reported improved compliance and reduced hysteresis, particularly with longer expiratory times. That study emphasised mechanical benefits, whereas our trial confirms clinical efficacy in a larger, randomised population. Differences in device type and ventilation setup (piston vs. turbine ventilator, linear flow release) may contribute to variation in observed outcomes, highlighting the importance of context‐specific application.

Given that FLEX ventilation appears to enhance oxygenation without requiring increased inspiratory pressures, it may offer a lung‐protective strategy for maintaining optimal pulmonary function without significant cardiovascular depression. This could be particularly advantageous in horses with pre‐existing respiratory compromise and those with haemodynamic instability, as FLEX has also been shown to reduce the level of PEEP requirement compared to VCV.^21^ Further studies are warranted to explore the long‐term effects of FLEX ventilation, its application in pathological lungs, and its potential to reduce peri‐anaesthetic complications. However, these results position FLEX ventilation as a significant advancement in equine anaesthetic management, offering a strategy that directly addresses the unique challenges of equine pulmonary physiology during general anaesthesia in dorsal or lateral recumbency. This study underscores the importance of species‐specific ventilation approaches and highlights the potential of innovative ventilatory techniques to enhance both anaesthetic safety and respiratory function in horses.

This study has several limitations that warrant consideration. First, no formal sample size calculation was performed prior to study initiation, which limits the ability to determine whether the study was adequately powered to detect differences in some secondary outcomes. However, the large sample size of over 400 horses minimises bias selection and increases the power of this study. Second, all horses included in this study were systemically healthy and underwent elective orthopaedic procedures; therefore, the findings may not be generalisable to horses with pre‐existing respiratory disease or undergoing emergency or non‐orthopaedic surgeries. Third, while ventilation mode was standardised, other potentially influential variables—such as the selection and dosage of vasopressors and inotropes, inhalant anaesthetics, and intraoperative fluid therapy—were left to the discretion of the attending anaesthesiologist. This lack of standardisation introduces clinical heterogeneity that may affect the reproducibility of our findings in different settings. Additionally, the non‐blinded nature of the study could have introduced bias in clinical management decisions, despite objective endpoints being used for analysis. Finally, FLEX ventilation was implemented using a specific ventilator and hardware setup, and it is unknown whether similar benefits would be observed using different equipment or in other equine populations. Future studies incorporating standardised cardiovascular support protocols and including systemically compromised or emergent cases are necessary to confirm the broader applicability and benefits of FLEX ventilation.

In conclusion, this study demonstrates the efficacy of FLEX ventilation in improving pulmonary gas exchange in anaesthetised horses compared to conventional VCV. FLEX significantly enhanced oxygenation, as evidenced by higher PaO_2_ and PaO_2_/FiO_2_, without PEEP or an alveolar manoeuvre. The benefits of FLEX on pulmonary function were more significant in dorsal than in lateral recumbent. These findings underline the potential of FLEX as a lung‐protective strategy that addresses species‐specific anaesthetic challenges, such as atelectasis and ventilation‐perfusion mismatch, by optimising expiratory flow dynamics. Future research should expand on these findings by investigating FLEX's applicability in compromised equine patients, its impact on long‐term outcomes, and its potential for reducing peri‐anaesthetic morbidity.

## FUNDING INFORMATION

The study was supported with intramural funding from the Department of Clinical Studies—New Bolton Center, University of Pennsylvania, School of Veterinary Medicine.

## CONFLICT OF INTEREST STATEMENT

The authors declare no conflicts of interest.

## AUTHOR CONTRIBUTIONS

**Klaus Hopster:** Conceptualization; investigation; funding acquisition; writing – original draft; methodology; validation; formal analysis. **Joao Henrique Neves Soares:** Conceptualization; investigation; funding acquisition; methodology; validation; writing – review and editing; supervision; formal analysis. **David Levine:** Conceptualization; investigation; methodology; writing – review and editing; formal analysis. **Kyla Ortved:** Conceptualization; investigation; methodology; writing – review and editing; formal analysis. **Bernd Driessen:** Conceptualization; investigation; methodology; validation; writing – review and editing; formal analysis; supervision. **Joaquin Araos:** Conceptualization; investigation; methodology; writing – review and editing; formal analysis; supervision.

## DATA INTEGRITY STATEMENT

Klaus Hopster had full access to all the data in the study and takes responsibility for the integrity of the data and the accuracy of data analysis.

## ETHICAL ANIMAL RESEARCH

This study was approved by the Institutional Animal Care and Use Committee (protocol # 806494) of the University of Pennsylvania.

## INFORMED CONSENT

Horse owners gave informed consent.

## Supporting information


**Figure S1.** Exemplary airway pressure and gas flow profile when using conventional volume‐controlled ventilation or flow‐controlled expiration ventilation modes in anaesthetised horses.

**Table S1:** Demographic and procedural characteristics of the study population (*n* = 406 horses). Values are presented as mean ± SD unless otherwise noted.

## Data Availability

The data that support the findings of this study are available upon reasonable request from the corresponding author. Open data sharing exemption granted by the editor.
