# Supplementary material for: Flow‐controlled expiration improves gas exchange in anaesthetised horses undergoing orthopaedic surgery
Source: Equine Vet J. 2025 Aug 21;58(4):917–24. doi: 10.1111/evj.70079 (PMC13244185; doi:10.1111/evj.70079)

Equine Veterinary Journal BEVA

Figure S1: Exemplary airway pressure and gas flow profile when using conventional volume-controlled ventilation or flow-controlled expiration ventilation modes in anaesthetised horses.

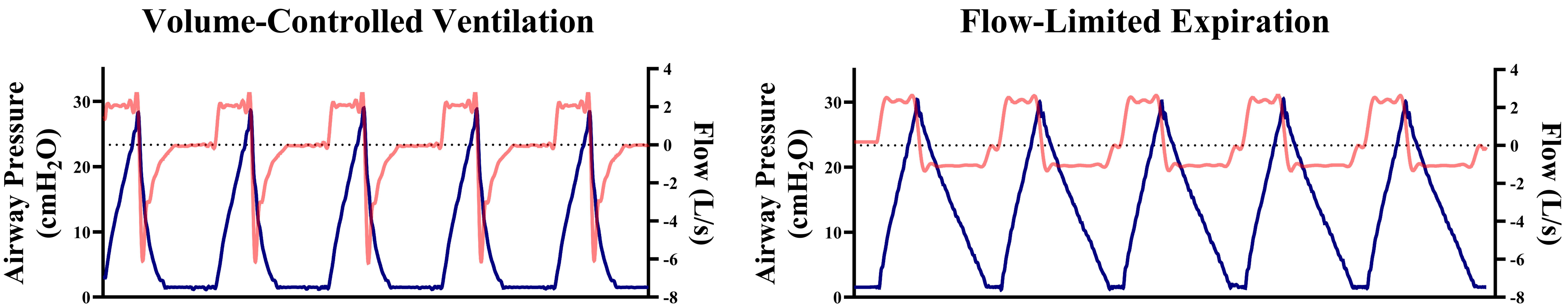

Supplement: Supplementary file 1 — Figure S1. Exemplary airway pressure and gas flow profile when using conventional volume‐controlled ventilation or flow‐controlled expiration ventilation modes in anaesthetised horses. [file EVJ-58-917-s002.pdf]
