# Supplementary material for: Flow‐controlled expiration improves gas exchange in anaesthetised horses undergoing orthopaedic surgery
Source: Equine Vet J. 2025 Aug 21;58(4):917–24. doi: 10.1111/evj.70079 (PMC13244185; doi:10.1111/evj.70079)
Supplement: Supplementary file 2 — Table S1: Demographic and procedural characteristics of the study population (n = 406 horses). Values are presented as mean ± SD unless otherwise noted. [file EVJ-58-917-s001.pdf]

Equine Veterinary Journal BEVA

Table S1: Demographic and procedural characteristics of the study population (n = 406 horses). Values are presented as mean ± SD unless otherwise noted.

**Characteristic**	**FLEX-D (n = 87)**	**VCV-D (n = 85)**	**FLEX-L (n = 115)**	**VCV-L (n = 119)**	**Total (n = 406)**
Age (years)	5 ± 4	6 ± 5	5 ± 4	7 ± 4	Median: 6 (Range: 3–19)
Bodyweight (kg)	477 ± 26	452 ± 37	485 ± 31	509 ± 24	Median: 482 (Range: 364–610)
Breed distribution					
Thoroughbred (%)	45 (52%)	42 (49%)	55 (48%)	60 (50%)	202 (50%)
Warmblood (%)	28 (32%)	29 (34%)	38 (33%)	41 (34%)	136 (33%)
Quarter Horse (%)	10 (11%)	9 (11%)	15 (13%)	13 (11%)	47 (12%)
Other (%)	4 (5%)	5 (6%)	7 (6%)	5 (4%)	21 (5%)
Sex					
Gelding (%)	45 (52%)	43 (51%)	65 (57%)	68 (57%)	221 (54%)
Mare (%)	35 (40%)	34 (40%)	40 (35%)	42 (35%)	151 (37%)
Stallion (%)	7 (8%)	8 (9%)	10 (9%)	9 (8%)	34 (8%)
Type of surgery					
Carpal arthroscopy (%)	20 (23%)	18 (21%)	28 (24%)	30 (25%)	96 (24%)
Fetlock arthroscopy (%)	22 (25%)	23 (27%)	32 (28%)	34 (29%)	111 (27%)
Stifle arthroscopy (%)	15 (17%)	14 (17%)	18 (16%)	20 (17%)	67 (17%)
Tarsal arthroscopy (%)	12 (14%)	11 (13%)	14 (12%)	15 (13%)	52 (13%)
Fracture repair (%)	10 (11%)	9 (11%)	15 (13%)	14 (12%)	48 (12%)
Other orthopaedic (%)	8 (9%)	10 (11%)	8 (7%)	6 (5%)	32 (8%)
